# Refolding of Cold‐Denatured Barstar Induced by Radio‐Frequency Heating: A New Method to Study Protein Folding by Real‐Time NMR Spectroscopy

**DOI:** 10.1002/anie.202006945

**Published:** 2020-09-25

**Authors:** György Pintér, Harald Schwalbe

**Affiliations:** ^1^ Institute for Organic Chemistry and Chemical Biology Center for Biomolecular Magnetic Resonance (BMRZ) Johann Wolfgang Goethe-Universität Frankfurt Max-von-Laue-Str. 7 60438 Frankfurt Germany

**Keywords:** barstar, proline isomerization, NMR spectroscopy, protein folding, temperature jump

## Abstract

The C40A/C82A double mutant of barstar has been shown to undergo cold denaturation above the water freezing point. By rapidly applying radio‐frequency power to lossy aqueous samples, refolding of barstar from its cold‐denatured state can be followed by real‐time NMR spectroscopy. Since temperature‐induced unfolding and refolding is reversible for this double mutant, multiple cycling can be utilized to obtain 2D real‐time NMR data. Barstar contains two proline residues that adopt a mix of cis and trans conformations in the low‐temperature‐unfolded state, which can potentially induce multiple folding pathways. The high time resolution real‐time 2D‐NMR measurements reported here show evidence for multiple folding pathways related to proline isomerization, and stable intermediates are populated. By application of advanced heating cycles and state‐correlated spectroscopy, an alternative folding pathway circumventing the rate‐limiting cis‐trans isomerization could be observed. The kinetic data revealed intermediates on both, the slow and the fast folding pathway.

## Graphical abstract

A state‐of‐the‐art temperature‐jump probe head is used in combination with real‐time 2D NMR experiments to study the kinetics of folding of a cold‐denatured protein. Barstar contains two prolines that adopt a mix of *cis* and *trans* conformations in the cold‐denatured state. The high time resolution measurements reported here show evidence for multiple folding pathways related to proline isomerization.

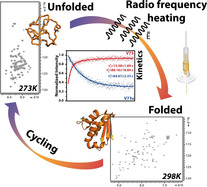

## Introduction

Understanding the driving forces of protein folding is a major focus of biophysics. Both, thermodynamic and kinetic studies of the different states, the unfolded and the folded state as well as intermediate states, are important. From first principles, the kinetics of protein folding involve numerous rate constants, potential accumulation of folding intermediates and exhibit distinct dependence on temperature, pressure, but also composition of solvents and solutes including chemical denaturants. The spectroscopic characterization of the kinetics of protein folding has been fascinating ever since protein folding has been conceptualized, allowing insights into intermediate states and activation energies along the folding pathway. Several theories have emerged based on experimental results ranging from stop‐flow measurement to protein engineering.^[1]^ Stopped‐flow measurements detected by fluorescence or circular dichroism have high time resolution but have limited structural resolution.

Nuclear magnetic resonance (NMR) spectroscopy stands out in its capability to characterize folding intermediates at atomic resolution. In NMR spectroscopy, there are two fundamentally different methods to investigate protein folding. Magnetization transfer experiments have been devised to study the kinetics of protein folding at equilibrium revealing information about excited or so‐called hidden states.[^2^, ^3^] These experiments can yield information about thermodynamics and kinetics of states involved in the folding. Protein folding can, however, also be studied under non‐equilibrium conditions.[^4^, ^5^, ^6^, ^7^, ^8^, ^9^] Methods most often induce folding from the unfolded to the folded state. The folding reaction is initiated either by chemical mixing,[^10^, ^11^] light induction[^7^, ^12^] or pressure‐jump. While the theory of T‐jump experiments is well described, only a limited number of NMR studies have been conducted thus far,[^13^, ^14^, ^15^, ^16^] and their time resolution has remained suboptimal. Temperature‐ and pressure‐jump kinetic folding experiments allow for multiple cycle experiments, provided the folding reaction is reversible. The release of high pressure of at least 1000 bar that unfolds a protein to an ambient pressure has been conceptualized for long[^17^, ^18^, ^19^] and recent technological developments have provided an outstanding benchmark for biophysical characterization utilizing the model protein ubiquitin.[^20^, ^21^, ^22^]

In the current study, we report on the development and application of a temperature jump (T‐jump) NMR to study the folding of the cold‐denatured barstar protein. By protein design, following literature, we achieved reversibility for the folding of barstar by mutating both cysteine residues of the protein (C40, C82) to alanine residues. By optimization of the technical aspects of the T‐jump probe, we achieved T‐jump for 20 °C in less than 500 ms. Within this time window, we minimize folding events during the dead time of the kinetic experiments to only 10 % of the slow phase of refolding. We thus demonstrate that our device combined with protein engineering and pulse‐sequence development opens up new avenues in time‐resolved protein‐folding studies.

Barstar shows both slow and fast folding pathways and populates several intermediates. The lifetime of these intermediates varies between microseconds to milliseconds and beyond a few minutes. Detailed thermodynamic analysis of different denatured states including heat‐, urea‐ and cold‐denatured states—and time‐resolved folding studies initiated from a urea‐ or guanidine‐hydrochloride‐denatured state have been reported.[^23^, ^24^, ^25^, ^26^, ^27^, ^28^, ^29^, ^30^, ^31^, ^32^, ^33^]

Three major steps are observed on the sequential refolding pathway of barstar: U→I_1_→I_2_→N. The first folding step occurs on a sub‐millisecond time scale and involves a structural collapse without secondary structure formation.[^26^, ^27^] Then, secondary structure forms in hundreds of milliseconds. This second intermediate lacks defined tertiary structures as its hydrophobic core binds ANS,^[26]^ nevertheless it shows already activity and binds barnase. The slowest step on the folding pathway involves the isomerisation of the Tyr47‐Pro48 peptide bond.[^24^, ^32^]

Here, we characterize the refolding of the cold‐denatured state by rapid heating in the NMR spectrometer using an in situ T‐jump setting^[34]^ and we apply double T‐jump experiments to study the alternative fast folding pathways of barstar from its cold‐denatured state.

## Results and Discussion

### Two Dimensional Kinetic Measurements with High Time Resolution

**Figure 1:**
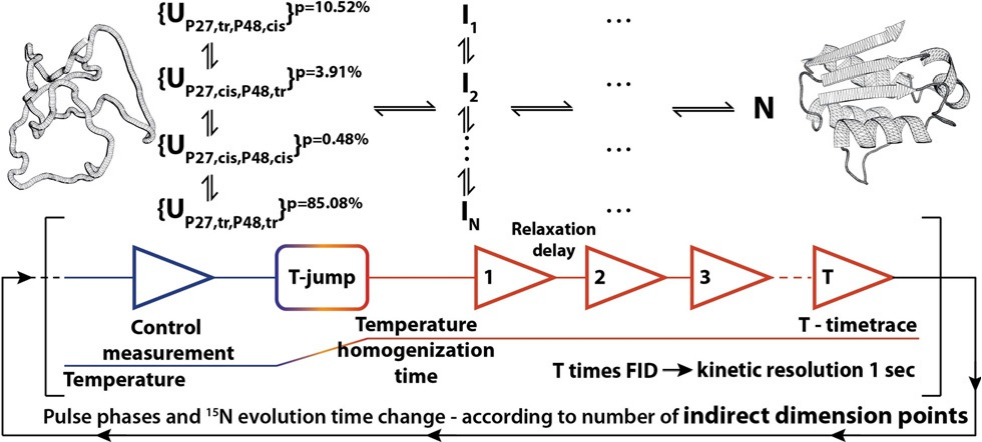
Schematic NMR acquisition for 2D T‐jump folding experiments. Between kinetic measurements (depending on the kinetic time frame for up to 510 FIDs are acquired over 10 minute timespan), the corresponding phases and ^15^N evolution time are incremented according States‐TPPI quadrature detection (usually kinetic experiments repeated 128 times). Post‐acquisition processing concatenates the corresponding time points into series of two‐dimensional spectra. The above part is an illustration to highlight that the possible starting conformations could lead to multiple folding intermediates, depending on the starting P27 and P48 conformation.

Recycling between the denatured and the folded state of a protein allows “infinite” repetition of the kinetic experiment. The requirement is that rapid heating and cooling of the sample are reversible. Wild‐type barstar contains two cysteine residues, C40 and C82. In previous folding studies of barstar, the cysteine‐to‐alanine double mutant was commonly used to avoid aggregation due to disulfide chemistry.[^24^, ^27^] This wild‐type‐like mutant shows no degradation or oligomerization even after several hundreds of refolding cycles (Supporting Information, Figure S3), allowing kinetic NMR acquisition schemes as indicated in Figure 1.

During the folding at given time points, the unfolded state U, the folded native state N and multiple intermediate states I_n_ can be present. In a simple case with a single intermediate we could theoretically observe the unfolded (U), the intermediate (I) and the native (N) state simultaneously with an expected low, unknown and high NMR signal dispersion, respectively. This leads to 267 possible cross peaks. The high resolution of two‐dimensional ^15^ N‐HSQC spectra allows to simultaneously resolve reporter signals for all of these states.

### Population of Different Conformational States of Proline Residues

**Figure 2:**
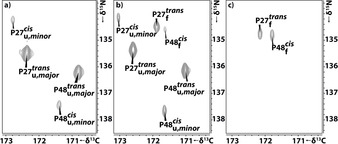
a) Proline‐selective^[37]^ CaCON‐type of spectra measured at a) 270 K, b) 288 K, c) 303 K from left to right, correlating C′_(i‐1)_‐N′_(i)(Pro)_ resonances in ω_2_ and ω_1_. a) In the unfolded (u) state both P27 and P48 residue adopt mixed *cis* and *trans* state, while in the folded (f) state c) P27 adopts *trans* conformation and P48 adopts the unusual *cis* conformation.

Applying the measurement scheme shown in Figure 1 with 10 minutes detection time and 30 minutes equilibration time resulted in 510 HSQC spectra. In the first spectra after the T‐jump, both folded and unfolded signals were observed (Supporting Information, Figure S1b and S9). Intensity changes during the kinetic time course could be followed for peaks from U and N. Barstar contains two proline residues. In the folded state, Leu26‐Pro27 has a *trans* amide bond conformation, while the Tyr47‐Pro48 amide bond is in *cis* conformation.[^35^, ^36^] To determine the population ratios of the possible conformations of the two proline residues in the cold‐denatured state, a proline‐selective carbon‐detected CaCON‐type experiment^[37]^ was measured showing that in the unfolded state there are four different conformations (Figure 2 a), while in the folded state only a single conformation exists (Figure 2 c), as both proline residues show a single major conformation. The population of the different conformations in the denatured state, by integrating the cross peaks, shows approximately that P27_*cis*_ 4.4 %, P27_*trans*_ 95.6 %, P48_*cis*_ 11 % and P48_*trans*_ is 89 %. The probability of the different starting conformations can be calculated as combinations of the populations (if we consider them independent) and the results are shown in Figure 1. The mixed population of the amide bonds already suggest that several pathways have to exist depending on the starting conformation. The major conformation of Tyr47‐Pro48 and the minor conformation of Leu26‐Pro27 has to undergo changes from *trans* to *cis* and *cis* to *trans*, respectively. According to literature, the *cis‐trans* proline isomerization occurs as the last step on the folding pathway, but intermediates for which proline isomerization has not occurred, are already functionally active and the secondary structure is similar to native state.

### Intermediate State—Double Exponential Kinetics

In the first HSQC spectrum after the T‐jump, signals from the unfolded state dominate Figure S9 in the Supporting Information, thus the unfolded state is predominantly populated. However, also native‐like signals are present.

**Figure 3:**
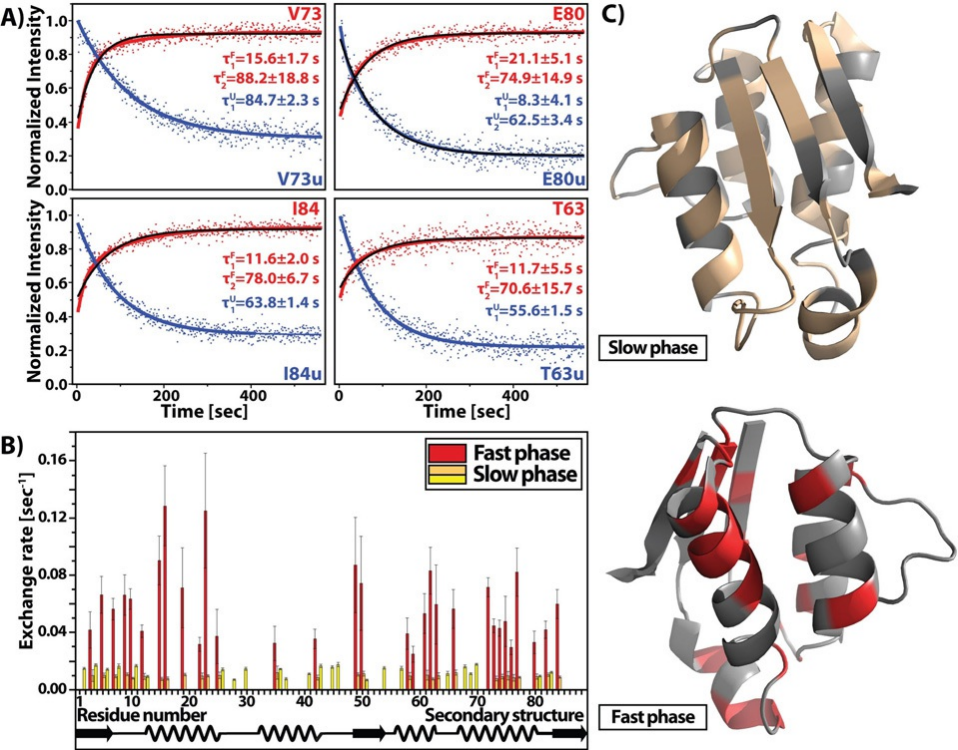
A) Representative time‐dependent signal intensities with double‐exponential decay fitting and corresponding fitting results. The measurements were done according to the pulse scheme in Figure 1 that resulted in a 510 HSQC time series, with signal‐to‐noise‐ratios ranging from 15:1 to 30:1. Color coding: red are signal intensity changes for signals of the folded state (N) blue for signals of the unfolded state (U), black line is mono‐exponential decay fit to show comparison. B) Bar diagram of the results of double‐ and mono‐exponential fits to the build‐up kinetics of signals from the folded state. The slow phase of the double‐exponential fit shows similar rates with mono‐exponential fits of other residues, except Q72 rate which resembles the faster part of the double‐exponential fits. C) Three dimensional structure of barstar (PDB: 1AB7)^[38]^ with residues colored to highlight slow (pale yellow) and fast (red) phase of observed kinetics of folded signals. For experimental details, see the Supporting Information.

Analyses of the kinetics observed on the folded and unfolded signals reveals single but also double‐exponential kinetics (Figure 3 A) for some signals. In principle, the kinetics are evident on every reporter signal, but the associated amplitude changes can differ for different reporter signals. Interestingly, only one (E80u) unfolded signal showed double‐exponential behaviour, while other unfolded signals can be fit by mono‐exponential decay (Supporting Information, Figure S6). Not all upcoming folded signals show the same behaviour. However, the slow phase of the kinetics fitted to double‐exponential build‐up typically compares well to the mono‐exponential rates (Figure 3 B).

### Stable Intermediate Signals

The time scale of the slower kinetic phase of the temperature‐induced refolding is of the order of 20 minutes. This is a suitable range to initiate the refolding not in a dedicated T‐jump probe head but by using the standard variable temperature unit of the NMR spectrometer. As this temperature unit is independent from the NMR probe head, we can utilize highest sensitivity probe heads to detect possible low intensity intermediates. Under these high sensitivity conditions, we recorded ^15^N‐SOFAST‐HMQC (SF‐HMQC) experiments; a single 2D experiment took 90 seconds. The measurement started when the sensor temperature reached target temperature.

**Figure 4:**
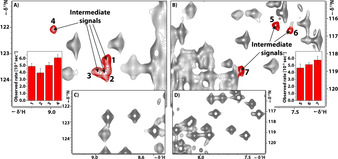
A and B) Different regions of SF‐HMQC spectra of barstar recorded after equilibrium cold denaturation at 278 K and the variable heating unit induced heating to 298 K—detected by the internal temperature sensor. Parts of the spectra are highlighted that show signals from folding intermediates. C,D) Identical spectral regions 20 minutes later. Bar diagrams, showing the observed decay rates of the intermediate signals.

Also, these experiments showed a high population of the unfolded state after reaching the final temperature in agreement with the spectra observed in T‐jump experiments. The data could be fitted to single exponentials, since the fast kinetic phase is lost during the longer dead time of the experiment. Nevertheless, this high signal‐to‐noise experiment yielded interesting information, as low intensity intermediate signals (Figure 4) were directly detectable, likely arising from a stable intermediate state. Signals show up already in the first spectrum and show decaying signal. The proton chemical shift dispersion indicates those intermediate signals to be native like. This observation suggests an already folded, native‐like structure is rapidly reached before the dead time of this heating experiment.

A plausible theory about the structural changes during the fast and slow phase of observed kinetics can be derived by mapping the residues with observed slow and fast phase kinetics on the structure (Figure 3 b and Figure 3 c) and reconciling literature data focusing on the structural changes of the folding process.[^26^, ^30^, ^31^, ^32^, ^33^] In the slow phase, as a last step, the final tertiary structure is consolidated as the directly involved residues of neighbouring secondary structural elements show intensity changes. The emergence of the final tertiary structure was also suggested by Fehrst^[24]^ and Udgaonkar.^[26]^ Residues which are involved in the fast folding phase (Figure 3 c) are mostly limited to parts of the sequence with defined secondary structure. Furthermore, the fast phase of kinetics almost completely avoids the binding site (residue 29–46 around helix_2_) of barstar and the correct folded signals can be observed, in agreement with previous studies showing activity of barstar before complete folding, as peptides with the binding site are also functional, albeit with reduced efficiency.^[39]^ Our data show that with different secondary structural elements fold at different time points during the folding process: helix_2_ for example shows no changes during the fast kinetic phase, while helix_4_ forms during the fast phase. These differences are hard to detect for many proteins for which all secondary structure is already formed in early intermediates and only tertiary structure formation occurs in late folding steps.[^40^, ^41^] Our data also support the mechanism of nucleation condensation suggested by Nölting et al. for barstar.^[42]^

### Characterizing the Fast Folding Pathway by “Double‐Jump” Heating Cycle

We further conducted double‐jump experiments because they are suitable to study the fast folding pathway of barstar by excluding the slow *trans‐cis* isomerisation of the Tyr47‐Pro48 amide bond. Thus, double‐jump experiments reduced the number of parallel folding pathways of barstar. The double jump is achieved by keeping the cooling time as short as possible. Recording a series of ^1^H experiments and analysing the line shape of the DSS reference signal (Supporting Information, Figure S7) showed that 2.5 minutes are sufficient to cool down the sample. From this non‐equilibrium denatured state that ensures the Tyr47‐Pro48 amide bond to predominantly adopt the *cis* conformation, we initiate the refolding step by T‐jump to follow the fast folding pathway. Comparison of the effect of the heating cycles (Supporting Information, Figure S1) show this heating cycle avoids the slow folding pathway.

### State‐Correlated Spectroscopy Between Cold‐denatured and Folded State

**Figure 5:**
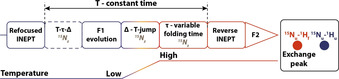
Schematic representation of a state‐correlating experiment and expected cross peaks. The applied pulse sequence was optimized to store magnetization on ^15^N_z_ spin order during longer delay times (Δ, *τ* and T‐*τ*‐Δ) to minimize the signal loss. The population ratio of the red cross peak (exchange peak) to the blue cross peak (still unfolded signal at the detection time point) depends on the *τ* folding time. The overall sensitivity mostly depends on the length of constant time “T”, albeit to increase S/N for shorter *τ* mixing times, the T‐*τ*‐Δ time can be set to zero for qualitative analyses.

We applied the double‐jump heating profile in combination with a new experiment, the pulse sequence scheme shown in Figure 5, to acquire information about the fast folding pathway. It is based on ^15^N‐ZZ‐exchange and state‐correlated experiments. Charlier et al. used a similar approach but initiating the magnetization transfer after P‐jump.[^20^, ^43^, ^44^] First, we transfer coherence at low temperature to the nitrogen. The t_1_ evolution takes place prior the T‐jump and thus, ^15^N chemical shifts are recorded from the unfolded state. After t_1_ evolution, the ^15^N_x_ coherence is converted to ^15^N_z_ to “store” ^15^N_z_ population on the slowest relaxing spin population, insensitive to temperature induced field inhomogeneities, during the T‐jump. After the sample reaches the final high temperature, further variable refolding times *τ* are implemented and finally ^15^N_z_ coherence is transferred back to ^1^H coherence and acquisition takes place. The direct dimension is acquired at high temperature and thus, ^1^H chemical shifts of the folded state are detected. The intensity changes of the exchange peaks as a function of *τ* report on the kinetics of the fast folding process and unambiguously link the various states involved in the folding process.

**Figure 6:**
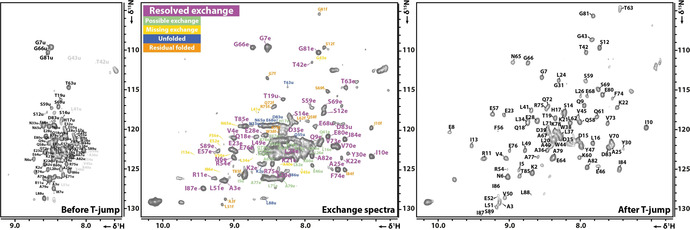
State‐correlating spectra recorded with the pulse scheme shown in Figure 5. Before and after T‐jump spectra were recorded without applying the radio frequency heating pulses while all other parameters are kept the same. This results in H,N‐correlation spectra with the same pulse sequence as the exchange spectra, similar to an HSQC spectrum but with same delays as in exchange spectra to show the effect of T1 relaxation. The exchange spectrum shown was recorded with a folding time *τ*=100 ms. Additional exchange spectrum with *τ*=5 ms and 200 ms can be found in Figure S8 in the Supporting Information.

The result of these experiments can be seen in Figure 6. The cross peaks show diverse intensity. By increasing the *τ* folding time from 5 ms to 100 ms and 200 ms exchange peak intensities increase and additional cross peaks become visible. From analysis of cross peaks, we could unambiguously detect intermediate states, as their intensities decayed for longer mixing times *τ*.

### Fast Folding Pathway with Intermediate

By analysing the time dependence of exchange experiments with different folding times (5 ms, 100 ms, and 200 ms) after T‐jump we observed unfolded peaks even after 200 ms. Some exchange peak (e.g. I84, I10, T85, A82) intensities are almost at maximum even with 5 ms folding time after T‐jump, while some exchange peaks (e.g. N6, I13, L41) are completely missing even with 200 ms folding delay. Control experiment with one second relaxation delay after the exchange experiment shows no unfolded peaks, concluding that the fast folding pathway takes no longer than two seconds. The most interesting peaks are from residues T63 and S69. The unfolded peaks of these residue are still observable (Supporting Information, Figure S2) at the shortest folding time experiment and completely vanished in 200 ms folding time experiment. The exchange peak intensities are increasing along with the folding time showing the ongoing folding process of barstar. These differences between the residues regarding observable unfolded and/or exchange peaks suggest that the folding is not uniform over the whole protein. It is interesting to discuss some aspects of the presence or absence of certain peaks. For example, signals for F56, I87, and S89 are not observable in the folded equilibrium spectra, presumably due to intermediate exchange, but I87 shows a clear exchange cross peak but not F56. Additional support for the non‐uniform folding kinetics comes from the observation of unfolded peaks which were not observable even in the shortest folding time experiment, while some remain visible even with 200 ms folding time. This is in agreement with literature described discharge temperature jump experiment.^[42]^ Nölting et al. showed by discharge T‐jump combined with fluorescence detection that there is a very short <4 ms change in the fluorescence of the unfolded state of barstar. They state this is due to the consolidation of hydrophobic interactions. Our results show that this observation is not the result of a complete loss of unfolded state, as some residues are still clearly observable, but there are also unfolded signals that are beyond detection even 5 ms after T‐jump.

The clear readout of intermediates along the fast folding pathway of barstar is achieved by experimentally focussing on a single of the multiple folding pathways. The unusual *cis*‐proline conformation of barstar in its native state and the mixture of *cis*‐*trans* populations in the cold‐denatured unfolded states allows for selective observation of the fast folding pathway. The double‐jump experiments start from high temperature with the peptide bond of Tyr47‐Pro48 in *cis* and of Leu26‐Pro27 in *trans*. Cooling down and thus preparing the cold‐denatured barstar results in a state where Tyr47‐Pro48 remains in *cis* and Leu26‐Pro27 in *trans* and thus populates a non‐equilibrium conformation for the cold‐denatured state of barstar. In the double‐jump experiments, the time prior to applying the second fast T‐jump is too short as to equilibrate the Tyr47‐Pro48 bond, and thus, refolding kinetics lack this phase. As a result, the spectra do not show the broadening observed in single T‐jump experiment due to state inhomogeneity. In the metastable state induced by double‐T‐jump, both proline bonds retain the native state conformation. Only after isolating a single out of multiple conformational unfolded substates allows detection of the intermediates states even starting from the non‐rate limiting and single *cis*‐Pro48 conformation, and thus shows the power of site‐resolved detection amenable to NMR spectroscopy.

With further technical improvement of the radio frequency heating, direct quantitative information about the kinetic could also be extracted. Currently, such quantification is limited by the T_1_ relaxation of the ^15^N_z_ magnetization, setting an upper time limit of the maximal pulse sequence length. Alternatively changing in our current probe head the nitrogen channel to carbon could also theoretically increase this time limit by utilizing the advantageous T_1_ relaxation time of the methyl groups. Reducing the dead time of the measurement would provide a more accurate zero time point, thus improve the possible time‐resolution.

## Conclusion

Cold‐denatured barstar is a suitable model system to study protein folding by T‐jump NMR experiment. The wild‐type‐like C40A/C82A double mutant can be reversibly refolded and in combination with the probe heads high stability and reproducibility allows recording of high‐quality two‐dimensional NMR experiments with high time resolution by cycling T‐jump experiments. Investigating the *cis‐trans* isomerization kinetics provided evidence by clear double‐exponential signal intensity change of intermediates. We could directly detect stable intermediate signals on the slow folding pathway. It showed that the major folding pathway has the rate limiting step of *trans‐cis* isomerization. Employing a double‐jump heating profile, insights into the fast folding pathway by a newly designed state‐correlated experiment could be obtained. The state correlated experiment revealed that the folding of barstar is not a simple downhill folding, but populates intermediate states as previously reported. The work reported here now resolves this kinetics site‐specifically observed. T‐double‐jump and P‐double‐jump experiments[^20^, ^45^] now allow for detailed delineation of intermediates along the different possible protein folding pathways that have been previously predicted but remained difficult to detect with site‐specific resolution.

## Conflict of interest

The authors declare no conflict of interest.

## Supporting information

As a service to our authors and readers, this journal provides supporting information supplied by the authors. Such materials are peer reviewed and may be re‐organized for online delivery, but are not copy‐edited or typeset. Technical support issues arising from supporting information (other than missing files) should be addressed to the authors.

Supplementary
