# Supplementary material for: Refolding of Cold‐Denatured Barstar Induced by Radio‐Frequency Heating: A New Method to Study Protein Folding by Real‐Time NMR Spectroscopy
Source: Angew Chem Int Ed Engl. 2020 Sep 25;59(49):22086–91. doi: 10.1002/anie.202006945 (PMC7756886; doi:10.1002/anie.202006945)
Supplement: Supplementary file 1 — Supplementary [file ANIE-59-22086-s001.pdf]

Angewandte Chemie
Eine Zeitschrift der Gesellschaft Deutscher Chemiker

Supporting Information

Refolding of Cold-Denatured Barstar Induced by Radio-Frequency Heating: A New Method to Study Protein Folding by Real-Time NMR Spectroscopy

*György Pintér and Harald Schwalbe**

anie_202006945_sm_miscellaneous_information.pdf

Table of Contents

Experimental Procedures **1**
Barstar preparation 1
Equilibrium NMR measurements 1
Kinetic NMR experiments 1
Results and Discussion **2**
Duty cycle improvement 2
Heating cycle comparison 2
State correlated spectra with different folding times 3
Comparison of folding cycle limits of wild-type and mutant barstar 3
VTU initiated refolding experiment 4
Exchange rates observed on unfolded signals 5
Minimum cooling time determination for double jump experiment 5
References **8**
Author Contributions **8**

Experimental Procedures

Barstar preparation

Wild-type barstar gene in pet3A vector was ordered from Genscript and was transformed into BL21 pLys S *E. Coli* strain (Promega). Expression and purification was done according to previously described method.[1] For ^{15}N and $^{15}N/^{13}C$ labelled proteins, cells have been grown in M9 media with ^{15}N-NH_4Cl and ^{13}C-glucose as a sole nitrogen and carbon source, for $^{2}H/^{15}N$ labelled proteins $^{2}H^{15}N$ labelled rich growth media for *E. coli* (Silantes) was used. As an additional step after the anion exchange chromatography another size exclusion chromatography step was introduced to exchange the sample into the final buffer condition and remove any residual contamination. Final NMR sample concentrations ranged between 1.5 to 3.0 mM barstar in 10 mM $K_xH_yPO_4$, 130 mM KCl buffer with additional 1.7-2.5 M urea. Additional 2.5 mM DSS was used as reference for chemical shifts and for internal temperature determination. All mutants of wild type barstar have been generated by site-directed mutagenesis with Agilent QuikChangeLightning kit according to manual protocol, the primers have been designed by QuikChange Primer Design (Agilent) and ordered from Eurofins Genomics.

Equilibrium NMR measurements

Equilibrium measurements were conducted on Bruker 600-900 MHz spectrometer with Avance 2-3 or Neon console equipped with cryogenically cooled triple resonance probehead. Assignment of cold denatured barstar has been done with routine BEST-TROSY type of HNCACB, HNcoCACB, HNCO, HNcaCO and hNcocaNNH measurements.[2] All pulse sequences were taken from Bruker standard library. Investigation of the proline residues conformational state has been measured by proline selective carbon detected CaCON type of experiment.[3]

Kinetic NMR experiments

Temperature jump experiments were measured on a Bruker 600 MHz spectrometer with a triple resonance probehead, with the internal coil designed to allow rapid heating of the sample. The probehead design and special processing program to combine the gas heating unit control and pulse sequence have been described in a previous paper.[4] Temperature jumps were optimized between 10-25°C resulting in 250 to 500 ms temperature jump times. 2D-HSQC series were measured by series of pseudo 2D experiments

and afterwards reconstituted by concatenating the same time points into the final spectrum. State-correlated experiments were measured the same way as 2D-HSQC and were reconstituted from series of pseudo-2D T-jump experiments. The number of scans were two and in the final reconstituted HSQC spectrum the signal-to-noise-ratio (S/N) is between 15-30 depending on the residue. S/N has been determined in Topspin by extracting one dimensional slices from the spectrum and using the "SINO real" function implemented in the software.

Experiments to measure *cis-trans* isomerization were initiated after one hour equilibration time at low temperature to reach equilibrium unfolded state (major conformation $Pro48_{trans}$) and during each T-jump experiments the sample was 20 minutes at high temperature. Between measurements 30 min cooling time was used. Measurements starting from denatured state with major conformation $Pro48_{cis}$ followed as: 20 minutes equilibration time at high temperature and short 2.5 min cooling time between T-jump experiments. All kinetic experiments were measured with the first 4 cycles used as dummy scans.

Results and Discussion

Duty cycle improvement

Duty cycle limits of the probehead setup was improved from 50% duty cycles with 300 W power amplifier to 90% by using 1000 W power amplifier.[4] This enabled faster temperature jumps, compared to the first description of the T-jump setup from 1 sec to 0.5 sec.

Heating cycle comparison

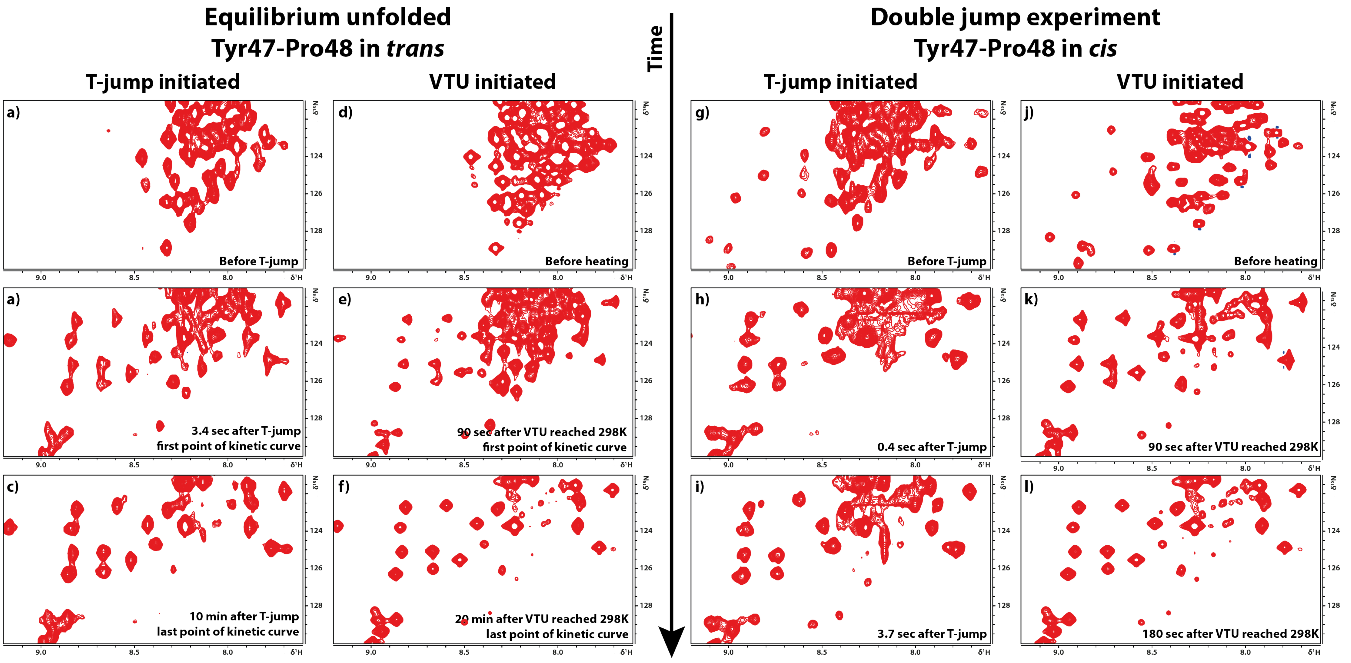

Figure S1. Time series and heating profile effect on the folding of barstar. Major conformation of Tyr47-Pro48 in equilibrium is trans while when cooling time is kept short in compared to the isomerization stays mostly in cis. This results in rapid refolding of barstar as is shown on the right side. 1-3 and 7-9 were measured with spectrometer equipped T-jump probehead with 64 points in indirect dimension and 4-6 and 10-12 were measured with spectrometer equipped with cryoprobe with 128 points in indirect dimension.

State correlated spectra with different folding times

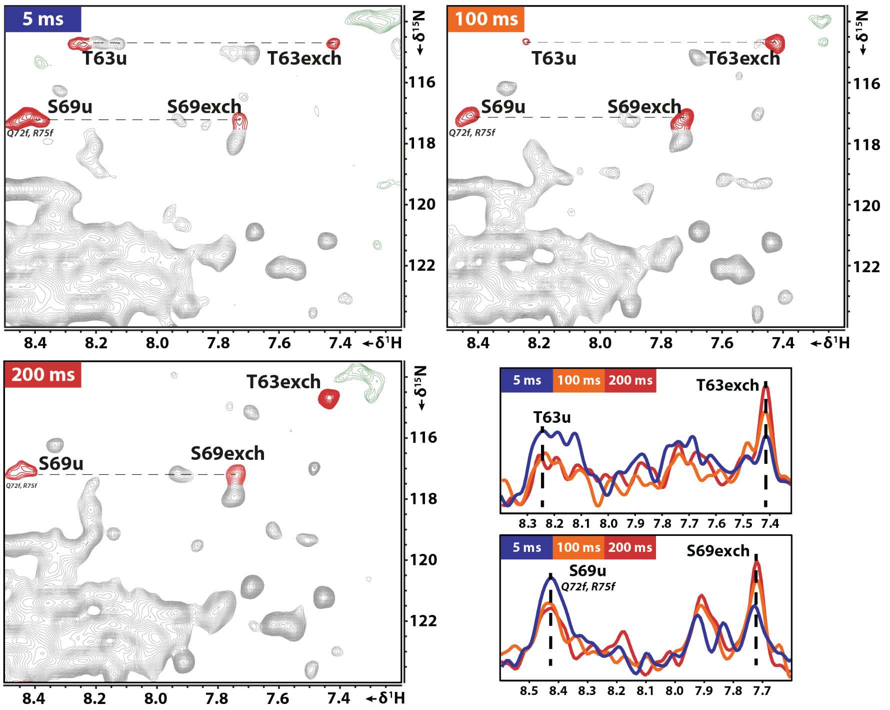

Figure S2. Series of state-correlated spectra with different τ folding time delays shown in upper left corner of each spectrum. Red cross-peaks show a pair of remaining unfolded and increasing exchange peak with increasing τ delay. Down right corner shows the projection overlays of the highlighted cross-peaks.

Comparison of folding cycle limits of wild-type and mutant barstar

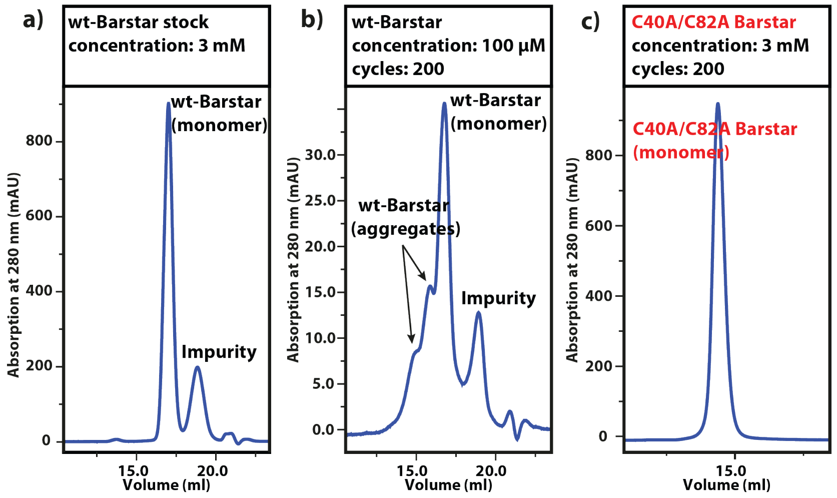

Figure S3. Size exclusion chromatograms of a) wt-barstar before thermocycling b) wt-barstar at 100 μM concentration after 200 cycles of 5 min at 4°C and 5 min at 30°C in standard thermocycler c) C40A/C82A double mutant Barstar after 200 cycles of 5 min at 4°C and 5 min at 30°C in standard thermocycler. The mutant did not show aggregation even at highest concentration used in T-jump experiments a) and b) were analysed on Superdex 200 column and c) was analysed on Superdex 75 Increase column.

VTU initiated refolding experiment

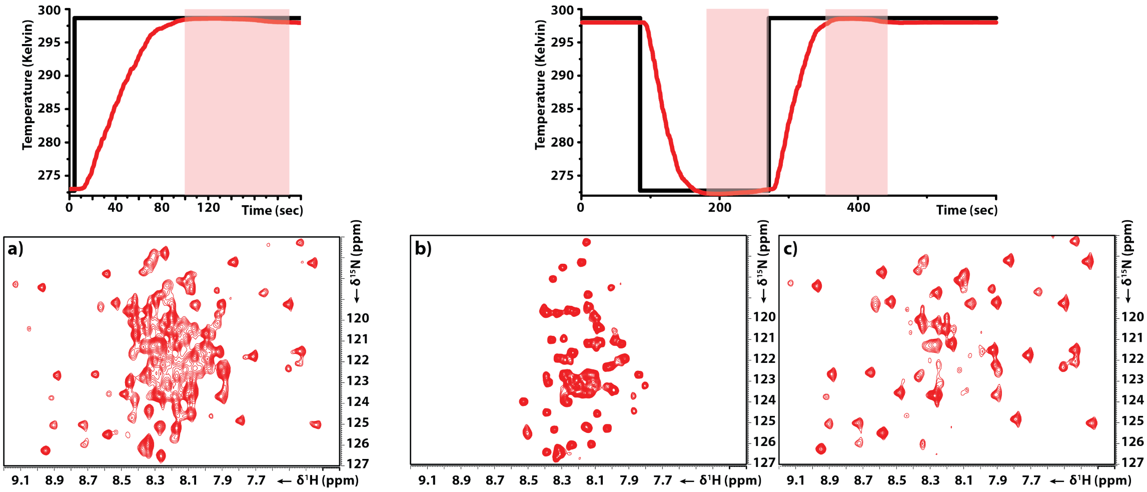

Figure S4. Folding initiated by using standard heating unit equipment. The upper figures show the corresponding heating unit target temperature (black) and sensor temperatures (red). Highlights on the time-temperature graphs show the timing of the experiments shown below. First SF-HMQC spectrum recorded after folding was initiated from a) equilibrium unfolded state and c) non-equilibrium unfolded states, b) shows non-equilibrium unfolded state.

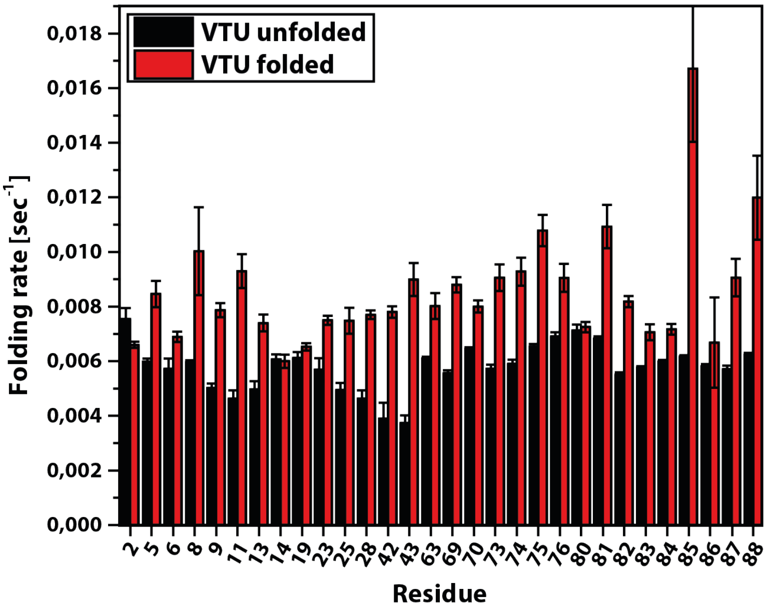

Figure S5. Bar diagram of folding rates of resolved peak pairs from mono exponential fit of time dependant signals. Black bar and red bar represent the same residue in the unfolded and folded state respectively. Measured by VTU heating experiment with a cryoprobe. Error bars represent fitting error.

Exchange rates observed on unfolded signals

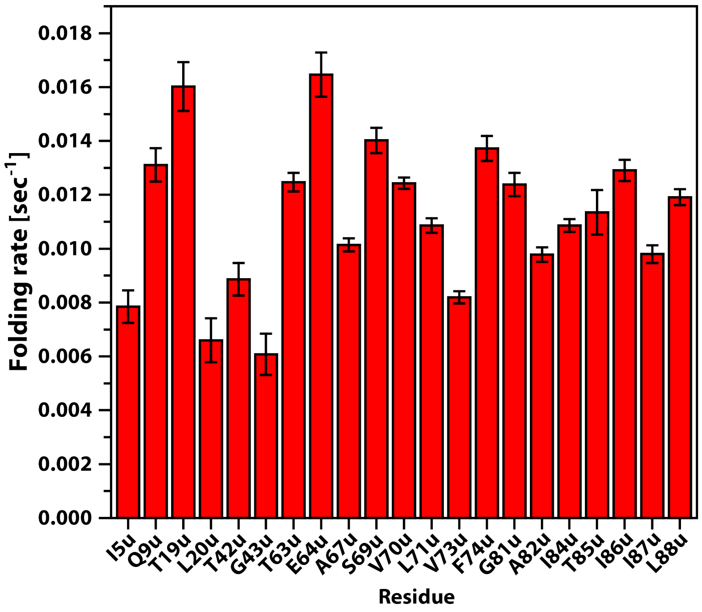

Figure S6. Bar diagram of exchange rates from mono-exponential fit of observed decaying time dependent signals of unfolded resolved signals from T-jump experiment. Error bars represent fitting error.

Minimum cooling time determination for double jump experiment

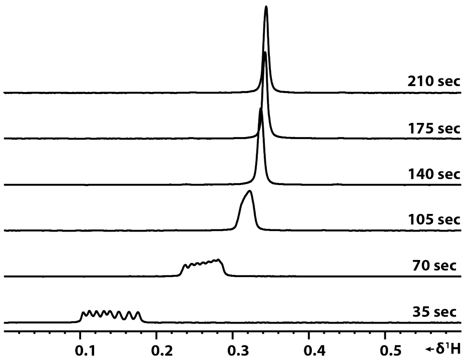

Figure S7. Proton spectra of DSS signal after starting the cooling process from 298K to 273K. Each spectrum was recorded with 8 scans, acquisition time of 1.7 seconds and relaxation delay of 1 second. No significant changes in linewidth or chemical shift after 140 second. DSS signal chemical shift changes due to the lock is fixed on the deuterium signal of the water.

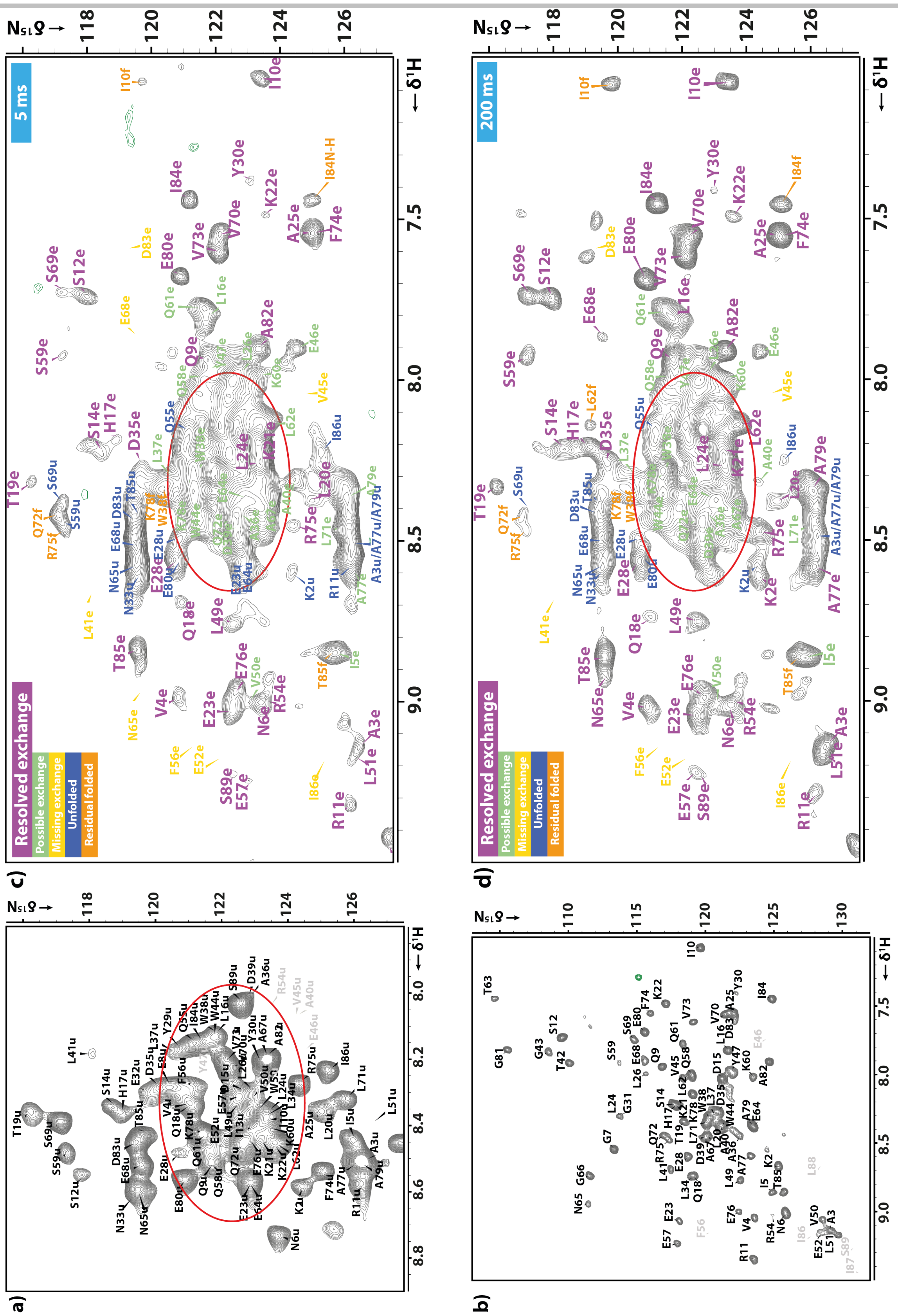

Figure S8. State-correlating spectra recorded with pulse scheme shown in Figure 5. a) Before and b) after T-jump spectra were recorded without applying the heating radio frequency pulses but otherwise unchanged parameters. This results in H,N-correlation spectra with the same pulse sequence as the exchange spectra. The exchange spectrum shown in c) was recorded with τ=5 ms and d) with τ=200 ms. Red ellipse in a-c) highlight the region which cannot be analysed due to the possible overlap of unfolded and exchange cross peaks in spectrum c) and d).

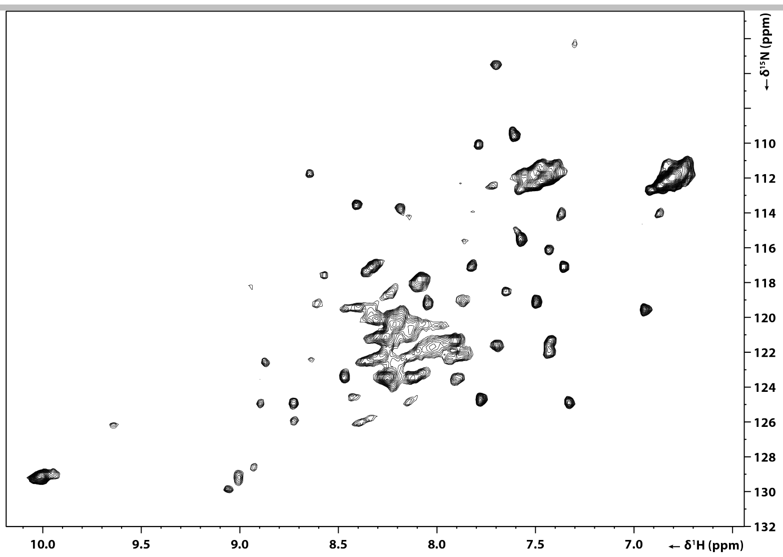

Figure S9. First time point of the long kinetic measurements. The measurement was started 100 ms after T-jump. Due to the temperature inhomogeneity the intensities of the spectrum were omitted from kinetic analyses. Major population is still unfolded as high signal intensity between 7.8 to 8.5 ppm, typical region of the unfolded signals, indicate, although some folded signals are already present.

Table S1. Statistical analyses of fitting time dependent signal intensities with mono- or biexponential functions. The fitting and statistical analyses have been done with Origin (OriginLab) software.

		K2	A3	I5	G7	E8	Q9
Akaike weight	MonoExp	0.734	0.006	0.000	0.000	0.438	0.000
	BiExp	0.266	0.994	1.000	1.000	0.562	1.000
Diff BIC	MonoExp	0.000	1.826	44.084	100.087	0.000	51.140
	BiExp	10.492	0.000	0.000	0.000	7.957	0.000
F-test	Prob>F	0.378	0.001	0.000	0.000	0.108	0.000
		I10	R11	S12	I13	D15	L16
Akaike weight	MonoExp	0.000	0.399	0.000	0.689	0.000	0.000
	BiExp	1.000	0.601	1.000	0.311	1.000	1.000
Diff BIC	MonoExp	172.953	0.000	54.868	0.000	67.856	60.019
	BiExp	0.000	7.636	0.000	10.044	0.000	0.000
F-test	Prob>F	0.000	0.092	0.000	0.303	0.000	0.000
		T19	K22	E23	A25	E28	Y30
Akaike weight	MonoExp	0.000	0.000	0.000	0.021	0.881	0.541
	BiExp	1.000	1.000	1.000	0.979	0.119	0.459
Diff BIC	MonoExp	10.481	37.233	23.684	0.000	0.000	0.000
	BiExp	0.000	0.000	0.000	0.775	12.457	8.783
F-test	Prob>F	0.000	0.000	0.000	0.003	1.000	0.162
		D35	A36	T42	G43	V45	E46
Akaike weight	MonoExp	0.044	0.524	0.000	0.865	0.881	0.763
	BiExp	0.956	0.476	1.000	0.135	0.119	0.237
Diff BIC	MonoExp	0.000	0.000	34.875	0.000	0.000	0.000
	BiExp	2.288	8.648	0.000	12.172	12.457	10.791
F-test	Prob>F	0.007	0.152	0.000	0.868	1.000	0.438
		L49	V50	L51	Q58	S59	Q61
Akaike weight	MonoExp	0.000	0.003	0.881	0.000	0.702	0.000
	BiExp	1.000	0.997	0.119	1.000	0.298	1.000
Diff BIC	MonoExp	11.836	3.410	0.000	11.116	0.000	10.878
	BiExp	0.000	0.000	12.457	0.000	10.170	0.000
F-test	Prob>F	0.000	0.000	1.000	0.000	0.322	0.000
		L62	T63	N65	G66	E68	S69
Akaike weight	MonoExp	0.000	0.008	0.847	0.000	0.881	0.678
	BiExp	1.000	0.992	0.153	1.000	0.119	0.322
Diff BIC	MonoExp	68.906	1.188	0.000	27.238	0.000	0.000
	BiExp	0.000	0.000	11.879	0.000	12.456	9.948
F-test	Prob>F	0.000	0.001	0.751	0.000	1.000	0.289
		V70	Q72	V73	F74	R75	E76
Akaike weight	MonoExp	0.324	0.450	0.000	0.000	0.003	0.000
	BiExp	0.676	0.550	1.000	1.000	0.997	1.000
Diff BIC	MonoExp	0.000	0.000	75.994	65.964	3.246	25.910
	BiExp	6.989	8.057	0.000	0.000	0.000	0.000
F-test	Prob>F	0.067	0.113	0.000	0.000	0.000	0.000
		A77	E80	G81	A82	D83	I84
Akaike weight	MonoExp	0.000	0.000	0.881	0.000	0.881	0.000
	BiExp	1.000	1.000	0.119	1.000	0.119	1.000
Diff BIC	MonoExp	65.894	21.977	0.000	77.848	0.000	85.080
	BiExp	0.000	0.000	12.457	0.000	12.457	0.000
F-test	Prob>F	0.000	0.000	1.000	0.000	1.000	0.000

References

[1] M. J. Lubienski, M. Bycroft, S. M. Freund, A. R. Fersht, *Biochemistry* **1994**, *33*, 8866–8877.
[2] Z. Solyom, M. Schwarten, L. Geist, R. Konrat, D. Willbold, B. Brutscher, *J Biomol NMR* **2013**, *55*, 311–321.
[3] W. Bermel, I. Bertini, J. Chill, I. C. Felli, N. Haba, V. K. M. V, R. Pierattelli, *ChemBioChem* **2012**, *13*, 2425–2432.
[4] J. Rinnenthal, D. Wagner, T. Marquardsen, A. Krahn, F. Engelke, H. Schwalbe, *Journal of Magnetic Resonance* **2015**, *251*, 84–93.
[5] E.-J. Wagenmakers, S. Farrell, *Psychonomic Bulletin & Review* **2004**, *11*, 192–196.

Author Contributions

G. Pintér conducted the research and analyzed the data, G. Pintér and H. Schwalbe designed the experiments, G. Pintér and H. Schwalbe contributed to manuscript writing.
